# Once-Daily Cyclosporine-A-MiDROPS for Treatment of Dry Eye Disease

**DOI:** 10.1167/tvst.7.5.24

**Published:** 2018-10-10

**Authors:** Terry G Coursey, Ronald A Wassel, Alexander B Quiambao, Rafal A Farjo

**Affiliations:** 1 EyeCRO, Oklahoma City, OK, USA

**Keywords:** dry eye disease, cyclosporine A, microemulsion, conjunctival goblet cells, small molecule, lipophilic eyedrop, MiDROPS

## Abstract

**Purpose:**

To determine if a Microemulsion Drug Ocular Penetration System (MiDROPS) formulation of cyclosporine A (CsA) delivers more drug and is more efficacious for treatment of dry eye disease (DED) than the current clinical formulation.

**Methods:**

Tissue distribution of CsA was quantified by liquid chromatography with tandem mass spectrometry (LC-MS/MS). To assess tolerability, CsA-MiDROPS (0.1%) was applied to the eyes of rabbits twice per day for 14 days and assessed using ophthalmoscopic examinations. Mice were exposed to desiccating stress for 10 days and received daily topical instillation of the vehicle or test agent. Cornea staining was done to quantify corneal permeability. Histologic quantification of goblet cell (GC) density and CD4^+^ T-cell infiltration in the conjunctiva was performed.

**Results:**

Ophthalmic distribution studies indicate significantly increased drug concentration with CsA-MiDROPS compared with Restasis. CsA-MiDROPS is well tolerated with little toxicity in a 2-week tolerability study. In the DED model, both 0.05% and 0.1% CsA-MiDROPS conferred a significant effect and were more effective than Restasis for treating experimental DED when dosed twice per day. As compared with Restasis dosed twice per day, 0.1% CsA-MiDROPS dosed once per day demonstrated superiority.

**Conclusions:**

CsA-MiDROPS showed superior drug delivery and efficacy compared with other clinical formulations. As this product is simple to produce and needs to be only applied once daily, the clinical development of CsA-MiDROPS will help to reduce societal and patient burdens by lowering drug costs and accelerating/improving the activity of CsA.

**Translational Relevance:**

MiDROPS has broad application concerning the ophthalmic development of lipophilic small molecule therapeutics.

## Introduction

Dry eye disease (DED) is a multifactorial syndrome that affects the daily lives of millions of people and is the result of dysfunctional tear production.^1^ In the United States alone, it is reported that up to 30% of the population over age 50 suffers from DED.^2^ Patients that suffer from DED have symptoms that often include eye irritation, redness, ocular discharge, decrease in tear volume, and can cause a significant decrease in the quality of life.^1,3,4^ Ocular surface pathology often includes superficial punctate lesions and epithelial defects. Over the last decade it has been established that DED is an inflammatory autoimmune disease, mediated primarily by CD4^+^ T cells.^5^ The initial induction of inflammation varies and can include systemic autoimmune diseases, such as Sjögren's syndrome, which cause destruction of the lacrimal gland, leading to aqueous deficient dry eye, or meibomian gland dysfunction, reducing the lipid component of the tear film, leading to evaporative dry eye.^1,6^ However, regardless of the initiating cause, the result is a loss of integrity of the tear film, leading to ocular surface inflammation.^7^

There are a number of nonpharmaceutical treatments for DED, ranging from artificial tears, punctal plugs, and autologous serum drops, all of which can offer temporary relief for mild cases of dry eye.^8,9^ There are currently only two Food and Drug Administration (FDA) approved drugs for treatment of DED. The most established and broadly used is Restasis (cyclosporine A [CsA] ophthalmic emulsion 0.05%; Allergan, Irvine, CA). CsA is an immunosuppressant that has also been used for the management of corneal graft rejection and autoimmune uveitis.^10^ Patients with severe DED frequently have a poor response to normal twice per day application of Restasis, even after several months of treatment, and often benefit from an increased dosing application of three or four times per day.^11^ It is thought that more frequent applications increase the therapeutic drug concentration in the conjunctiva and cornea, which improves the outcome through more effectively reducing inflammation by controlling inflammatory mediators, such CD4^+^ T cells. However, with increased application requirements, the rate of patient compliance typically decreases. Therefore, a better system for drug delivery that increases the concentration of CsA within the conjunctiva and cornea, could lead to dramatic improvements in the clinical outcome for patients with moderate to severe DED. CsA is notoriously difficult to formulate in conventional topical eye drops due to its hydrophobic structure and very poor solubility in water. The limitations of the current FDA-approved treatment afford an opportunity for the development of improved formulations that will provide for increased available drug concentration while ensuring safety and tolerability.

To enable formulation of lipophilic small molecules, such as CsA, we developed a novel and patented ocular drug delivery platform called Microemulsion Drug Ocular Penetration System (MiDROPS; Patent numbers- US-8,968,775 and US-9,149,453). This system produces microemulsions with droplets that are typically less than 10 nm in diameter, thermodynamically stable, and optically transparent. Although microemulsions contain higher levels of surfactants than classical emulsions, we have previously demonstrated that several MiDROPS formulations are well tolerated upon exaggerated dosing to large animals and can deliver efficacious amounts of test agent into tissues of the eye.^12,13^ MiDROPS can elicit superior effects compared with conventional eye drops because microemulsions provide for enhanced absorption of the drug in the cornea and conjunctiva and can prolong drug release; thus, increasing drug concentrations within ocular tissues for sustained periods of time. This platform has allowed a fundamental shift of what drug concentrations can be achieved and the types of materials can be used during topical instillation, as long as they are prepared within the correct system. While CsA has been difficult to formulate into efficacious and stable eye drops, we identified a number of parental formulations in the MiDROPS library suitable for clinical development.

**Figure 1:**
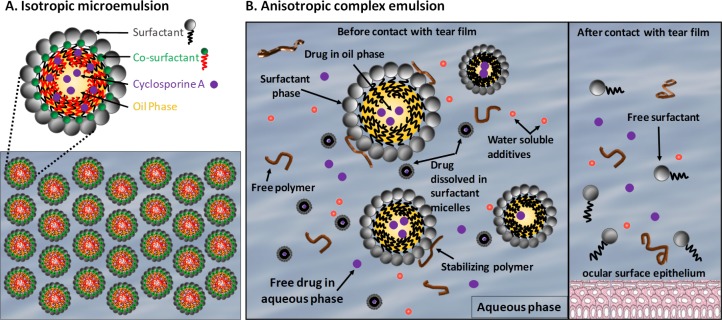
Isotropic microemulsions versus anisotropic complex emulsions. (A) Isotropic microemulsions, such as CsA-MiDROPS, consist of single phase where the drug is contained within the microemulsion. As microemulsions are dilutable, there is not a breakdown of structure when exposed to the tear film. (B) Anisotropic complex emulsions, such as Restasis, consist of many phases. When exposed to the tear film the emulsion breaks down exposing free surfactants to the ocular surface.

Herein, we demonstrate that CsA-MiDROPS is capable of safely delivering substantially higher amounts of CsA into the eye, as compared with current clinical formulations. This enhanced delivery correlated with increased activity of CsA to ameliorate corneal permeability and conjunctival damage and inflammation that occur in a mouse model of DED. In addition, CsA-MiDROPS has distinct advantages over complex emulsions, such as Restasis, because they are dilutable isotropic microemulsions that do not break down when exposed to the tear film. Such breakdown leads to the release of free surfactants that may cause irritation to the ocular surface^14^ (Fig. 1B). With complex emulsions like Restasis, the CsA is present in multiple phases and could possess a different biological activity in each phase, which may limit the effective delivery of CsA to the affected tissues.^14^ Additionally, MiDROPS are thermodynamically stable with a potentially long shelf life, spontaneously form under the correct conditions, and are easy and cheap to manufacture, in stark contrast to the complicated manufacturing process of other complex CsA emulsions.^14^ The availability of a more efficacious, better tolerated, and cost-effective formulation of CsA could improve the lives of millions of patients that suffer from DED.

## Methods

### Animals

Eight-week-old C57BL/6J female mice were purchased from The Jackson Laboratory (Bar Harbor, ME). We have chosen to use female mice for the DED studies, as there is increased severity of disease and are more representative of the human patient population.^15,16^ Pharmacokinetics studies were performed in 8- to 10-week-old female C57BL/6J mice and female 3- to 4-month-old Dutch-belted rabbits (Robinson Services, Inc., Mocksville, NC). Female 3- to 4-month -old New Zealand White rabbits (Covance Research Products, Princeton, NJ) were used for tolerability studies. The Institutional Animal Care and Use Committee at the University of Oklahoma Health Sciences Center have approved all animal experiments. All studies adhered to the ARVO Statement for the Use of Animals in Ophthalmic and Vision Research.

### Induction of DED in Mice

DED (desiccating stress [DS]) was induced by subcutaneous injection of scopolamine hydrobromide (0.5 mg/0.2 mL; Sigma-Aldrich, St. Louis, MO) four times a day (08:00, 11:00, 14:00, and 17:30 hours) for five consecutive days (DS5) or 10 consecutive days (DS10), as previously described.^17–20^ Mice were placed in a cage with a perforated screen on both sides to allow airflow from a fan placed 6 inches in front of it. Room humidity and temperature was maintained at 20 ± 3% and 25 ± 3°C in a specifically designed highly controlled environmental chamber. Control mice were maintained in a nonstressed (NS) environment at 55 ± 3% relative humidity without exposure to a forced air draft. Animals received drug treatment from the beginning of the experiment.

### Corneal Permeability

Corneal epithelial permeability to Oregon Green Dextran (OGD; 70,000 molecular weight; ThermoFisher, Waltham, MA) was assessed by instilling 0.5 μL of OGD onto the ocular surface 1 minute before euthanasia, as previously described.^20–22^ Corneas were rinsed with balanced saline solution (BSS) and photographed under fluorescence excitation at 470 nm. The severity of corneal OGD staining was graded in digital images in the 2-mm central zone of each cornea by two masked observers, using NIS Elements software (Nikon, Tokyo, Japan). To account for between-eye correlation the average of both eyes were plotted as a single data point.

### Histology, Periodic Acid Schiff Staining, and Conjunctival Goblet Cell Density Measurement

Enucleated mouse eyes were fixed in 10% formalin, and embedded in paraffin. Eight micrometer sections were stained with Periodic acid-Schiff (PAS) reagent, as previously described.^20,21^ Goblet cell (GC) density in the superior and inferior conjunctiva was measured in three or four eyes per group using NIS Elements Software.

### CD4 Immunohistochemistry

Immunohistochemistry was performed to detect and count the number of cells in the conjunctival epithelium that stained positively for CD4 (clone H129.9, 2 μg/mL; BD Bioscience, San Jose, CA) and anti-rat IgG biotinylated secondary antibody (2 μg/mL goat anti-rat, 559286; BD Pharmingen, San Jose, CA)/Vectastain Elite ABC (using NovaRed reagents; Vector, Burlingame, CA), as previously described.^21,22^ Secondary antibody alone and proper anti-mouse isotype (BD Biosciences) controls were also performed. Positively stained cells were counted in the goblet cell rich area of the conjunctiva using NIS Elements Software.

### Toxicology Assessment

Hackett-McDonald scoring was used to assess corneal clarity, fluorescein staining, vascularization, hyperemia, and chemosis of the ocular surface. Full ocular examinations via biomicroscopy assessed the iris, aqueous flare, vitreous cell, and pupil response. Indirect dilated ophthalmoscopy evaluated the lens, vitreous clarity and hemorrhage, retinal detachment and hemorrhage, and choroid/retina inflammation. Blood samples were collected in order to monitor systemic CsA exposure.

### Sample Preparation and LC-MS/MS Analysis

#### Sample Preparation

Target tissue was weighed in individual MP BIO FastPrep tubes. Garnet Matrix A lysis beads (MP BIO, Santa Ana, CA) were added to each tube followed by 300 μL of water. Tissues were homogenized for two 40 second runs with a MP BIO FastPrep 96 homogenizer. The samples were then centrifuged at 18,000*g* for 5 minutes. Two hundred microliters of this homogenate was combined with 10 μL of internal standard (ketoprofen; 10 μg/mL) and mixed. Samples were then loaded on to a HyperSep supported liquid extraction (SLE) 96-well plate (ThermoFisher). Samples were allowed to filter through the plate by gravity alone and sit for 10 minutes. One pound per square inch of positive pressure (nitrogen gas) was applied for 5 seconds to remove any excess liquid in the plate. To extract the compounds of interest from the SLE plate, 1.5 mL of methyl-tert butyl ether (MTBE) was used. The MTBE was evaporated under gently flowing nitrogen. The samples were then reconstituted with 200 μL of methanol and were ready for LC/MS analysis.

#### LC-MS/MS Analysis

Analysis was performed using an API 4000 mass spectrometer (AB Sciex, Framingham, MA) coupled to a Nexera X2 UHPLC system (Shimadzu Corporation, Kyoto, Japan). The analytical column was a Thermo Accucore C18 column 2.6 μm (50 × 2.1 mm). Mobile phase A consisted of 0.1% formic acid dissolved in deionized water, and mobile phase B consists of 0.1% formic acid dissolved in methanol. The analysis was performed using a simple, rapid gradient increasing from 35% B to 95% B over 2 minutes, holding at 95% B for 2 minutes, and then re-equilibrating at the initial 35% B for 2 minutes between injections. The flow rate was 300 μL per minute and the column temperature was maintained at 60°C. Multiple reaction monitoring (MRM) of cyclosporine A was performed using electrospray in positive ion mode. The MRM transition was 1202 → 425. The source was operated at 400°C with an electrospray voltage of 5500 V. Ion source parameters were as follows: curtain gas 25 L/min, GS1 60, GS2 45, CAD gas 10 L/min.

### Statistical Analysis

Sample size and power calculations were performed using Statmate software. Statistical analyses were conducted with GraphPad Prism software (GraphPad, La Jolla, CA). Data were first evaluated for normality with the Kolmogorov-Smirnov (KS) normality test. Appropriate parametric (*t*-test) or nonparametric (Mann-Whitney *U* or Wilcoxon) statistical tests were used to make comparisons between two groups. ANOVA or the Kruskal-Wallis tests were used to evaluate the data. Student's *t*-test was used to assess significance between two groups. ANOVA analysis followed by the post hoc Tukey (Fisher's protected least significant difference) was used to assess statistical significance between multiple groups. *P* ≤ 0.05 was regarded as statistically significant. Power analysis was used to calculate the minimum number of animals to be used in each experiment.

## Results

### MiDROPS Formulation Screen to Identify CsA-MiDROPS Formulation Candidates

**Figure 2:**
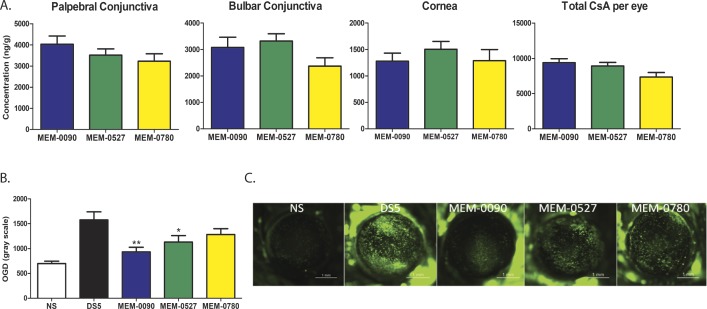
Screening of MiDROPS library yielded three potential CsA-MiDROPS candidates. (A) Dutch-Belted rabbits were treated with 50 μL of 0.1% CsA-MEM-0090, 0.1% CsA-MEM-0527, or 0.1% CsA-MEM-0780 twice per day for 9 days. Three animals (6 eyes) per group were harvested for determination of CsA concentration by LC-MS/MS. (B) C57BL/6 mice were exposed to desiccating stress (DS) for 5 days in all groups except NS. The NS group was not exposed to DS. Corneal permeability staining was performed using OGD on the morning of the fifth day. Animals were treated twice per day with 2 μL per eye of 0.1% CsA-MEM-0090, 0.1% CsA-MEM-0527, or 0.1% CsA-MEM-0780. *P > 0.05; **P > 0.005. (C) Representative images of cornea staining in each group. Bar graphs show mean ± SEM. A total of 10 mice were examined per group. *P > 0.05; **P > 0.005 compared with DS5.

The MiDROPS platform allows for the screening of over 1000 different parental formulations to find the best fit microemulsion formulation for a particular hydrophobic compound. We conducted a screen based upon microemulsion formation with 0.1% CsA, which yielded 11 parental formulation candidates. These formulations were scaled up and evaluated according to droplet size, viscosity, and stability for 2 weeks at 25°C and 4°C. Eight formulations passed these acceptance criteria (Table 1). To narrow down our candidate list we performed an ocular distribution screen with these formulations in topical instillation to C57BL/6 mice. Mice were dosed twice per day (BID) for 5 days and CsA concentrations in ocular tissues were determined by LC-MS/MS. Three formulations, CsA-MEM-0090, CsA-MEM-0527, and CsA-MEM-0780, delivered the highest amounts of CsA to murine ocular tissues (data not shown). To confirm these results, we conducted a follow-up ocular distribution where Dutch-Belted rabbits were treated with 50 μL of 0.1% CsA-MEM-0090, 0.1% CsA-MEM-0527, or 0.1% CsA-MEM-0780 BID for 9 days. Three animals per group were examined. All three formulations showed similar delivery of CsA to the palpebral conjunctiva, bulbar conjunctiva, and cornea (Fig. 2A). We then performed a pharmacology study where C57BL/6 mice were exposed to DS for 5 days and treated BID with 2 μL of 0.1% CsA-MEM-0090, 0.1% CsA-MEM-0527, or 0.1% CsA-MEM-0780. Ten animals per group were examined. After 5 days the corneas were stained with OGD to quantify corneal permeability.^23,24^ While both 0.1% CsA-MEM-0090 and 0.1% CsA-MEM-0527 conferred a significant reduction in corneal permeability as compared with the DS5 control, the 0.1% CsA-MEM-0090 had significantly better results than the others and was chosen for further study (Fig. 2B). Throughout the remainder of this study CsA-MEM-0090 is referred to as CsA-MiDROPS.

**Table 1:**
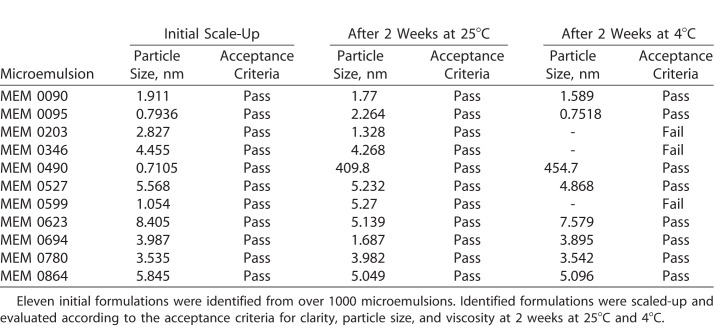
Results of High Throughput MiDROPS Screen Using 0.1% CsA

### Significantly Increased CsA Concentration With CsA-MiDROPS Compared With Restasis

**Figure 3:**
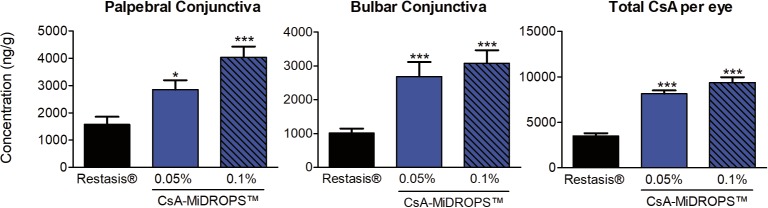
Significantly increased CsA concentration with CsA-MiDROPS compared with Restasis. Dutch-Belted rabbits were treated with 50 μL per eye twice per day with Restasis (0.05% CsA), 0.05% CsA-MiDROPS, or 0.1% CsA-MiDROPS for 9 days. Three animals (6 eyes) per group were harvested for determination of CsA concentration by LC-MS/MS. Bar graphs show mean ± SEM. *P > 0.05; ***P > 0.0005 compared with Restasis.

To determine the CsA concentration delivered by CsA-MiDROPS compared with Restasis in ocular tissues, the eyes of pigmented Dutch-Belted rabbits were treated with 50 μL of Restasis, 0.05% CsA-MiDROPS, or 0.1% CsA-MiDROPS BID for 8 days. Three animals per group were examined. On the morning of the ninth day animals received one dose and ocular tissues were harvested and dissected 1 hour after instillation to determine of the amount of CsA present by LC-MS/MS. CsA concentration in the palpebral conjunctiva, bulbar conjunctiva, and cornea was 2 to 3 times higher in eyes treated with either 0.05% or 0.1% CsA-MiDROPS as compared with Restasis (Fig. 3 and Table 2). Because immune responses that are elicited upon induction of dry eye often occur in the conjunctiva, higher bioavailability of CsA should dampen the chronic inflammatory response seen in dry eye. These results suggest that the MiDROPS platform significantly improves drug delivery of CsA to the conjunctiva and cornea. CsA was not detected in blood plasma, suggesting that CsA-MiDROPS treatment is localized to ocular tissues and has minimal systemic exposure (Table 2). In addition to tissues in the ocular surface, CsA was found in equivalent or increased concentrations in other ocular tissues (Table 2).

**Table 2:**
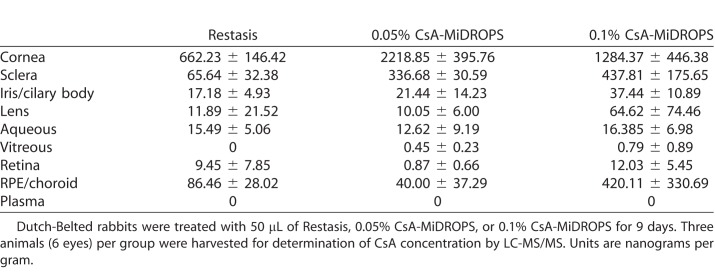
CsA Ocular Tissue Concentrations in PK Studies

As seen in Table 2 and Supplementary Figure S1, 0.05% yielded better delivery to the cornea than the higher concentration of 0.1%. Although not completely understood, we speculate that changing the drug concentration may slightly alter the structure of the microemulsion and change drug delivery to specific tissues, such as the cornea, and different tissues may respond differently. Tissue specific responses to drug delivery by MiDROPS deserve further examination. We made an effort to present the whole data set of CsA concentrations within ocular tissues. With respect to the RPE/choroid data, this was a small sampling (*n* = 3 rabbits/6 eyes total per treatment), and the value reported for 0.1% CsA-MiDROPS was skewed by a couple of data points leading to a large SEM (420.11 ± 330.69; Table 2 and Supplementary Fig. S1). We are currently performing a number of rigorous ophthalmic pharmacokinetic studies in rabbits and dogs to confirm these results.

Overall, delivery in other tissues is as expected and our main point of emphasis is that we are delivering greater amounts of CsA to the palpebral conjunctiva, resulting in increased therapeutic activity as this is the site of disease-mediating CD4^+^ T cell infiltration.

### CsA-MiDROPS is Well Tolerated

Considerable tolerability issues have been reported with Restasis.^25^ In order to assess tolerability, 50 μL of CsA-MiDROPS (0.1%) was applied to the eyes of New Zealand White rabbits BID for 13 days and one dose on the 14th day. Baseline examinations were conducted prior to the start of the study and on study days 7 and 14. Hackett-McDonald scoring (0–4) was used to assess corneal clarity, fluorescein staining, vascularization, hyperemia, and chemosis of the ocular surface.^26^ Full ocular examinations by biomicroscopy assessed the iris, aqueous flare, vitreous cell, and pupil response. Indirect dilated ophthalmoscopy was used to assess the lens, vitreous clarity and hemorrhage, retinal detachment or hemorrhage, and choroid/retina inflammation. Little to no ocular surface irritation was observed (Table 3). Three animals per groups were examined.

**Table 3:**
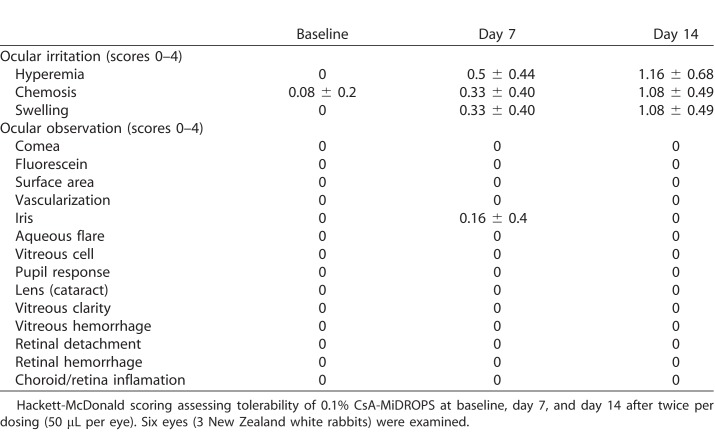
CsA-MiDROPS are Well Tolerated

### Once-Daily CsA-MiDROPS (0.1%) is Superior to Twice Per Day Restasis in the Induced Dry Eye Model

In order to compare the efficacy of CsA-MiDROPS with that of Restasis for treatment of DED, C57BL/6 mice were exposed to DS for 10 days to allow accumulation of CsA into ocular tissues. Mice were divided into nine treatment groups with 10 animals randomly assigned per group. As a negative control, one group of mice was NS and housed at a fixed room humidity of 55%; and as a positive control, another group was exposed to DS for 10 days without drug treatment (DS10). Animals were treated from start of the experiment with 2 μL Restasis BID or with 2 μL 0.05% CsA-MiDROPS, 0.1% CsA-MiDROPS, or MiDROPS vehicle BID or once daily (QD). After 10 days the corneas were stained with OGD to quantify corneal permeability.^23,24^ Ten animals per group were examined. All corneas were assessed for corneal permeability on the morning of the tenth day.

**Figure 4:**
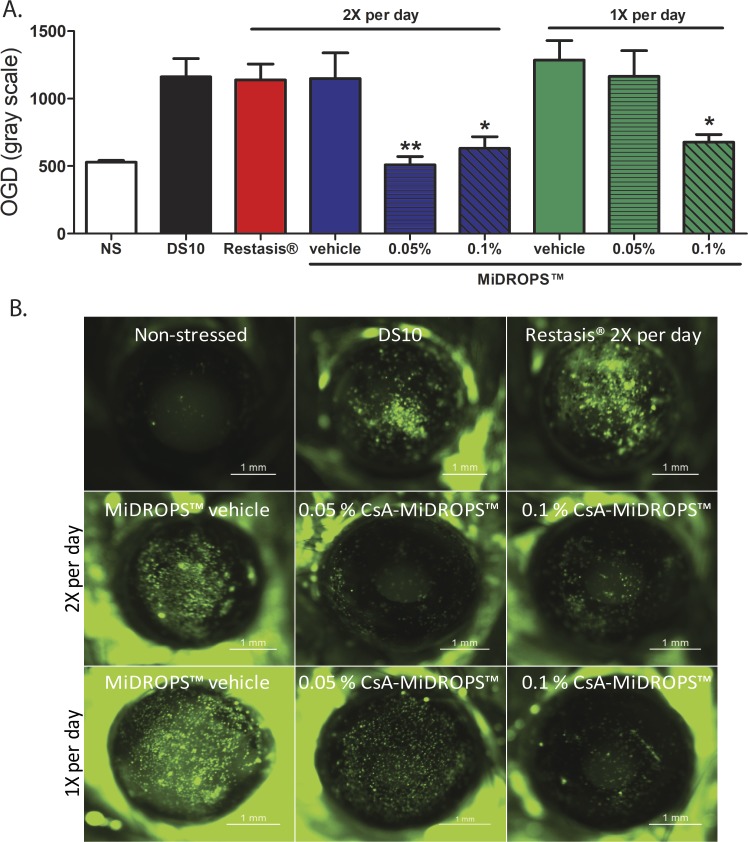
QD 0.1% CsA-MiDROPS significantly reduces corneal permeability staining compared with twice per day Restasis. C57BL/6 mice were exposed to DS for 10 days in all groups except NS. The NS group was not exposed to DS. Corneal permeability staining was performed using OGD on the morning of the tenth day. (A) Animals were treated twice per day with Restasis (red bar), MiDROPS vehicle, 0.05% CsA-MiDROPS, or 0.1% CsA-MiDROPS (blue bars). Mice were also treated one per day with MiDROPS vehicle, 0.05% CsA-MiDROPS, or 0.1% CsA-MiDROPS (green bars). All dosing was 2 μL per eye. (B) Representative images of cornea staining in each group. A total of 10 mice were examined per group. Bar graphs show mean ± SEM. *P > 0.05; **P > 0.005 compared with DS10, Restasis, or MiDROPS vehicle.

In this study, BID Restasis was not effective at reducing corneal permeability compared with the DS10 control (Fig. 4). This is consistent with published reports showing that Restasis does not reduce corneal permeability staining, but does reduce epithelial apoptosis.^27^ We suggest that the effectiveness of Restasis is severely limited by low concentration of CsA delivered to ocular tissues over this 10-day study. Treatment with Restasis would likely reduce dry eye parameters with increased dosing volumes or application frequency. In contrast, animals treated BID with either 0.05% or 0.1% CsA-MiDROPS had significantly less corneal staining than the DS10 control and BID Restasis. Both concentrations of CsA-MiDROPS with BID application significantly reduced corneal permeability and were not significantly different (*P* = 0.267) from each other suggesting this may be maximum effective concentration needed to treat the disease. There was a significance difference between BID and QD treatment with 0.5% CsA-MiDROPS (*P* = 0.0005), but not between BID and QD treatment with 0.1% CsA-MiDROPS (*P* = 0.536). When CsA-MiDROPS were dosed QD, we observed a reduction in corneal permeability with the 0.1% formulation, but not with the 0.05% formulation, that was similar to the maximal effect we observed of the 0.1% formulation when applied BID (Fig. 4). The *P* value comparing QD 0.1% and 0.05% was *P* = 0.041. We also examined the MiDROPS vehicle (without CsA), which did not produce a significant change in corneal permeability with application QD or BID. Overall, these results suggest that QD 0.1% CsA-MiDROPS was more effective than BID Restasis in reduction of corneal permeability in this induced DED model.

A key parameter in monitoring the immunoregulatory activity of CsA is determination of the CD4^+^ T cell activity in the conjunctiva. CD4^+^ T cells have been shown to mediate DED causing both corneal permeability and loss of conjunctival goblet cells.^5,21,22,28,29^ Conjunctival goblet cell loss occurs in patients with DED and can be reduced in patients with extended use of CsA.^30,31^ It has been demonstrated that CD4^+^ T cells mediate conjunctival goblet cell apoptosis via production of IFN-γ.^29,32,33^ Thus, conjunctival goblet cell loss is a key additional parameter in the determination of the efficacy of a DED treatment.

**Figure 5:**
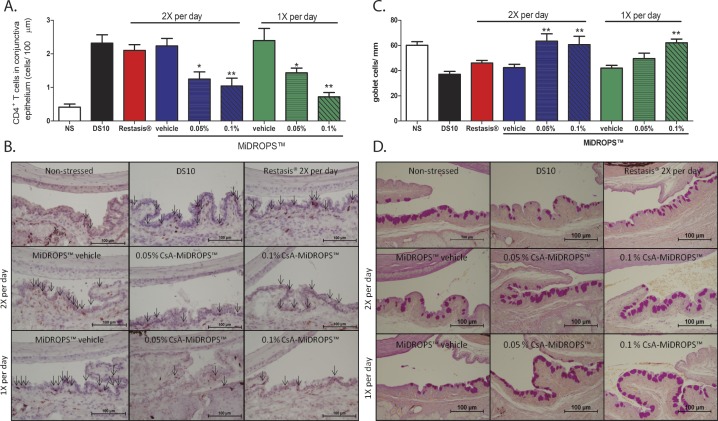
CsA-MiDROPS inhibits CD4^+^ T cell infiltration and loss of conjunctival GC compared with Restasis. C57BL/6 mice were exposed to DS for 10 days in all groups except NS. The NS was not exposed to DS. Eyes were collected for histologic analysis for CD4^+^ T cell density or conjunctival GC density. Animals were treated twice per day with Restasis (red bar), MiDROPS vehicle, 0.05% CsA-MiDROPS, or 0.1% CsA-MiDROPS (blue bars). Mice were also treated once daily with MiDROPS vehicle, 0.05% CsA-MiDROPS, or 0.1% CsA-MiDROPS (green bars). (A) CD4^+^ T cell density (B) Representative ×20 images of CD4 staining in each group for CD4 T cell quantification. Arrows denote cell with positive staining. (C) Conjuntival GC density. (D) Representative ×20 images of PAS staining in each group for goblet cell density quantification. A total of three to four eyes (6–8 conjunctivas) were analyzed per group. Bar graphs show mean ± SEM. *P > 0.05; **P > 0.005 compared with DS10, Restasis, or MiDROPS vehicle.

In order to examine the ability of CsA-MiDROPS to regulate inflammatory responses, we determined CD4^+^ T cell and GC density in the conjunctiva. Our results suggest that animals treated QD or BID with 0.1% CsA-MiDROPS had significantly less CD4^+^ T cell infiltration and GC loss than the DS10 control and BID Restasis. Animals treated with 0.05% CsA-MiDROPS had significantly less CD4^+^ T cell infiltration with either QD and BID dosing. There were not significant differences (*P* = 0.566) between BID 0.05% versus BID 0.1% CsA-MiDROPS; however, there was (*P* = 0.004) comparing QD 0.05% versus QD 0.1% CsA-MiDROPS. There were not significant differences between BID and QD treatments of CsA-MiDROPS at the same concentration (*P* = 0.427 for BID versus QD 0.05% CsA-MiDROPS and *P* = 0.295 for BID versus QD 0.1% CsA-MiDROPS; Figs. 5A, 5B).

Prevention of GC loss was observed with BID application, but not with QD application of 0.05% CsA-MiDROPS (Figs. 5C, 5D). The MiDROPS vehicle (without CsA) did not produce a significant change with application QD or BID for either CD4^+^ T cell or goblet cell density (Fig. 5). Although it has been reported that CsA reduces conjunctival GC loss in animal models,^27^ we did not observe a significant difference compared with the DS10 control (*P* = 0.674; Figs. 5C, 5D), which again might be due to poor delivery of CsA to the conjunctiva by Restasis. A significant difference was not observed between BID 0.05% and 0.1% treatments (*P* = 0.797). QD treatment with 0.1% CsA-MiDROPS significantly reduced GC loss compared with QD 0.05% CsA-MiDROPS (*P* = 0.038). There were not significant differences between BID and QD treatments of CsA-MiDROPS at the same concentration (*P* = 0.088 for BID versus QD 0.05% CsA-MiDROPS and *P* = 0.852 for BID versus QD 0.1% CsA-MiDROPS).

Overall, our results suggest that we are able to deliver two to three times more CsA to ocular tissues with CsA-MiDROPS than Restasis, while maintaining ocular tolerability. This increased delivery with a QD treatment of 0.1% CsA-MiDROPS was able to significantly reduce corneal permeability, CD4^+^ T cell infiltration, and GC loss compared with a BID treatment with Restasis.

## Discussion

Here, we demonstrate increased delivery of CsA to ocular tissues with our novel MiDROPS platform. Via a high throughput screen of over 1000 formulations we were able to identify formulations that spontaneously form microemulsions and are most suited for that particular hydrophobic small molecule. Using this technology, we were able to achieve CsA concentrations two to three times greater than what is delivered by the leading clinical formulation of CsA for ophthalmic use, Restasis. We suggest that this increased delivery is responsible for the increased efficacy of CsA-MiDROPS. Even with significantly increased concentrations of CsA to ocular tissues, we did not observe increased tolerability issues, suggesting that the tolerability issues with Restasis may be more related to the formulation rather than CsA itself.

A direct comparison in the murine experimental DED model between BID 0.05% CsA-MiDROPS and BID Restasis (0.05% CsA) suggests CsA-MiDROPS is superior for reduction of corneal permeability, CD4^+^ T cell infiltration and conjunctival GC loss. BID treatment with 0.1% CsA-MiDROPS did not further reduce disease parameters suggesting that BID 0.05% CsA-MiDROPS achieves the maximal effect of CsA for treatment of DED. QD treatment with 0.1% CsA-MiDROPS significantly reduces corneal permeability, CD4^+^ T cell infiltration, and conjunctival GC loss compared with BID Restasis. This suggests that QD treatment with CsA-MiDROPS could be superior to BID Restasis in treatment of patients with DED and may increase patient compliance and reduce medication costs. QD 0.05% CsA-MiDROPS did not reduce corneal permeability and goblet cell loss compared with Restasis; however, QD 0.05% CsA-MiDROPS did significantly reduce CD4^+^ T cell infiltration suggesting that the level of CsA is approaching the therapeutic threshold needed to effectively reduce other disease parameters. Overall, these results suggest that CsA-MiDROPS is superior to Restasis in this experimental DED model.

Restasis is often an effective treatment for many patients that suffer from DED. However, in many cases of moderate to severe DED, the CsA drug bioavailability is not sufficient to inhibit the inflammation that causes tear instability in the conjunctiva and cornea. CsA is a very hydrophobic molecule with a high LogP, making it difficult to formulate in aqueous solutions. While Restasis offers clinical benefit, there are substantial tolerability issues in at least 17% of patients who exhibit “ocular burning” following topical instillation.^25^ Restasis is formulated with 0.05% CsA in a complex emulsion of glycerin (2.2%), castor oil (1.25%), polysorbate 80 (1.00%), carbomer copolymer type A (0.05%), purified water (to 100%), and sodium hydroxide for pH adjustment.^25^ Effective treatment using Restasis requires application at least BID, however some patients reportedly only benefit from four to five applications per day, creating a burdensome eye drop regimen, decreased compliance and more frequent side effects.^11^

We suggest that tolerability issues are likely caused by the formulation, as complex emulsions have free surfactants and free drug in the aqueous phase, which could damage the cornea and induce pain^14^ (Fig. 1B). Our novel CsA-MiDROPS microemulsions do not contain free surfactants and the drug substance is only present inside or at the surface of the oil core, which may alleviate tolerability issues. A significant increase in the available drug concentration at the ocular surface could provide a substantial improvement in clinical symptoms in patients with moderate to severe DED with fewer applications. In addition to these benefits, a QD treatment regimen would decrease the cost to the patient and increase patient compliance.

Restasis is a complex emulsion with components distributed in various phases determined by the their physicochemical properties as well as the process used for manufacturing the emulsion^14^ (Fig. 1B). For example, surfactants partition into the water phase to form micelles, in addition to acting as an emulsifier to stabilize oil droplets. Oil droplets form globules with a range of sizes (z-average sizes are reported to be 183.93 ± 0.72 to 659.30 ± 25.08 nm).^34^ CsA, dissolved in oil droplets, partitions into other phases, such as the water phase, micellar phase, microemulsion phase, or at the oil/water interface.^14^ Thus, CsA is expected to be present in Restasis simultaneously in several different phases, including (1) in the aqueous phase, (2) in micellar equilibrium in the aqueous phase (in both the micellar core and in the surfactant palisade layers), (3) in the oil droplets, (4) in the surfactant monolayer of the oil/water interface, and (5) associated with viscosity agents.^14^ CsA may have a different biological activity in each phase, and each phase may have different ocular tolerability properties. This suggests that only a fractional percentage of the 0.05% CsA may penetrate tissues and exact an immunomodulatory effect. Furthermore, the tolerability of each phase is unknown.

In contrast to complex emulsions, CsA-MiDROPS is a clear, thermodynamically stable, isotropic liquid mixture of oil, water, and surfactants. This Winsor type IV microemulsion represents a single phase where droplet sizes range between 2 and 8 nm. As there is only one phase, the activity and tolerability properties of CsA remain consistent throughout the microemulsion (Fig. 1A). In general, microemulsions are quite similar to micelles. They are optically transparent, have low viscosity, and comprise thermodynamically stable dispersions of oil and water stabilized by an interfacial film of a surfactant (usually in combination with a co-surfactant). There are three types of microemulsions, water-in-oil (W/O), bicontinuous, and oil-in-water (O/W).^35^ The type of microemulsion is largely dependent on the ratio of oil to water. When the continuous phase comprises oil, a W/O microemulsion is formed. When the continuous phase is comprised of water with oil droplets suspended in it, an O/W microemulsion is formed. The combination of surfactant and co-surfactant at the correct ratio to oil and water leads to a spontaneous formation of the microemulsion and a reduction in size (<50 nm) of the droplets constituting the internal phase. The chemical and structural properties of the drug determine the type of microemulsion and the composition of surfactant/co-surfactant. Utilization of a large library of formulations allows for the tailoring of a specific drug to the correct formulation, thereby yielding improved drug delivery to ocular tissues.

In clinical trials, the most common adverse reaction following the use of Restasis was ocular burning in 17% of patients.^25^ We suspect that a majority of this pain is likely due to the presence of free surfactant on the cornea. Prior to administration, free surfactant is present in the formulation, but as it mixes with the tear film, the emulsion is destabilized, freeing up additional surfactant that could interact with the cornea epithelium.^14^ Thus, microemulsions are better tolerated on the ocular surface due to a lack of free surfactants. Importantly, microemulsions are dilutable in aqueous substrates and do not break down when exposed to the corneal tear film interface. While having a high amount of surfactant (between 30% and 40% in some cases) in the formulations, the surfactant molecules are ordered in the emulsion droplets in such a way that the cornea is only exposed to the hydrophobic component of the surfactant and thus no free surfactants are expected to interact with the cornea.^14^

Microemulsions are easy to manufacture and sterilize and use a straightforward production process. In addition, CsA-MiDROPS are thermodynamically stable, which can potentially confer a longer shelf-life than complex emulsions. Microemulsions are formed by spontaneous self-assembly following the correct combination of components. Whereas complex emulsions require a complicated three-step process to produce.^14^ Using the MiDROPS system, we are easily able to formulate a thermodynamically stable isotropic formulation of CsA with increased delivery to ocular tissues and potentially fewer side effects. CsA-MiDROPS can be easily produced in a multidose package or single-use vials.

Overall, these results suggest that the clinical development of a QD treatment with our CsA formulation using the MiDROPS platform is well tolerated and could be equally or more efficacious than the currently available CsA formulation for treatment of DED.

## Supplementary Material

Supplement 1
