# Supplementary material for: Once-Daily Cyclosporine-A-MiDROPS for Treatment of Dry Eye Disease
Source: Transl Vis Sci Technol. 2018 Oct 10;7(5):24. doi: 10.1167/tvst.7.5.24 (PMC6181191; doi:10.1167/tvst.7.5.24)

**Supplementary Figure 1: Scatter plots of CsA drug distribution in the cornea and RPE/ Choroid of the data found Table 2.** Dutch-Belted rabbits were treated with 50 µl of Restasis®, 0.05% CsA-MiDROPS™, or 0.1% CsA-MiDROPS™ for nine days. Three animals (six eyes) per group were harvested for determination of CsA concentration by LC-MS/MS. Units are ng/g.

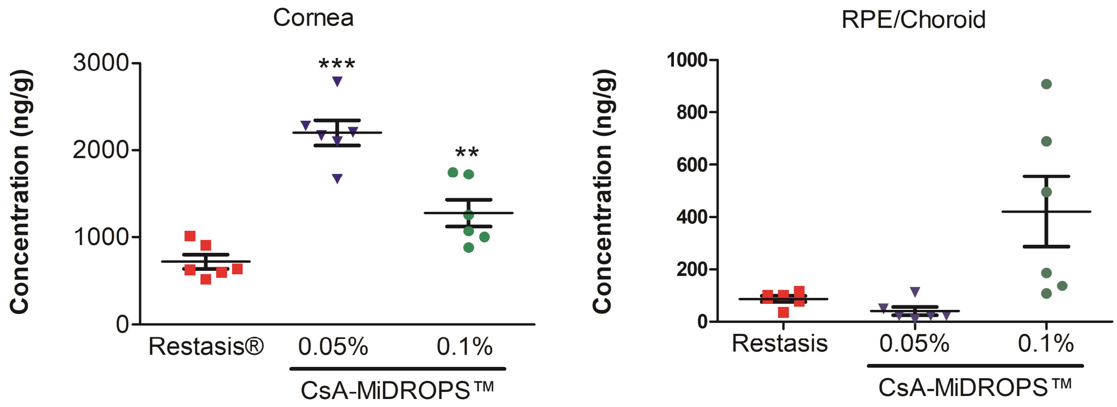

Supplement: Supplement 1 [file tvst-07-05-17_s01.pdf]
